# An integrative profiling of metabolome and transcriptome in the plasma and skeletal muscle following an exercise intervention in diet-induced obese mice

**DOI:** 10.1093/jmcb/mjad016

**Published:** 2023-03-07

**Authors:** Shuang Han, Qingqian Wu, Mengying Wang, Miqi Yang, Chen Sun, Jiaqi Liang, Xiaozhen Guo, Zheyu Zhang, Jingya Xu, Xinyuan Qiu, Cen Xie, Siyu Chen, Yue Gao, Zhuo-Xian Meng

**Affiliations:** Department of Pathology and Pathophysiology and Department of Cardiology of the Second Affiliated Hospital, Zhejiang University School of Medicine, Hangzhou 310058, China; Department of Geriatrics, Affiliated Hangzhou First People's Hospital, Zhejiang University School of Medicine, Hangzhou 310006, China; Department of Pathology and Pathophysiology and Department of Cardiology of the Second Affiliated Hospital, Zhejiang University School of Medicine, Hangzhou 310058, China; Key Laboratory of Disease Proteomics of Zhejiang Province, Zhejiang University School of Medicine, Hangzhou 310058, China; Department of Big Data in Health Science School of Public Health, Zhejiang University School of Medicine, Hangzhou 310003, China; Department of Pathology and Pathophysiology and Department of Cardiology of the Second Affiliated Hospital, Zhejiang University School of Medicine, Hangzhou 310058, China; State Key Laboratory of Natural Medicines and School of Life Science and Technology, China Pharmaceutical University, Nanjing 211198, China; State Key Laboratory of Natural Medicines and School of Life Science and Technology, China Pharmaceutical University, Nanjing 211198, China; State Key Laboratory of Drug Research, Shanghai Institute of Material Medical, Chinese Academy of Sciences, Shanghai 201203, China; Department of Pathology and Pathophysiology and Department of Cardiology of the Second Affiliated Hospital, Zhejiang University School of Medicine, Hangzhou 310058, China; Department of Pathology and Pathophysiology and Department of Cardiology of the Second Affiliated Hospital, Zhejiang University School of Medicine, Hangzhou 310058, China; Department of Biology and Chemistry, College of Liberal Arts and Sciences, National University of Defense Technology, Changsha 410073, China; State Key Laboratory of Drug Research, Shanghai Institute of Material Medical, Chinese Academy of Sciences, Shanghai 201203, China; State Key Laboratory of Natural Medicines and School of Life Science and Technology, China Pharmaceutical University, Nanjing 211198, China; Department of Geriatrics, Affiliated Hangzhou First People's Hospital, Zhejiang University School of Medicine, Hangzhou 310006, China; Department of Pathology and Pathophysiology and Department of Cardiology of the Second Affiliated Hospital, Zhejiang University School of Medicine, Hangzhou 310058, China; Department of Geriatrics, Affiliated Hangzhou First People's Hospital, Zhejiang University School of Medicine, Hangzhou 310006, China; Key Laboratory of Disease Proteomics of Zhejiang Province, Zhejiang University School of Medicine, Hangzhou 310058, China

**Keywords:** type 2 diabetes, exercise intervention, metabolomics, transcriptomics

## Abstract

Exercise intervention at the early stage of type 2 diabetes mellitus (T2DM) can aid in the maintenance of blood glucose homeostasis and prevent the development of macrovascular and microvascular complications. However, the exercise-regulated pathways that prevent the development of T2DM remain largely unclear. In this study, two forms of exercise intervention, treadmill training and voluntary wheel running, were conducted for high-fat diet (HFD)-induced obese mice. We observed that both forms of exercise intervention alleviated HFD-induced insulin resistance and glucose intolerance. Skeletal muscle is recognized as the primary site for postprandial glucose uptake and for responsive alteration beyond exercise training. Metabolomic profiling of the plasma and skeletal muscle in Chow, HFD, and HFD-exercise groups revealed robust alterations in metabolic pathways by exercise intervention in both cases. Overlapping analysis identified nine metabolites, including beta-alanine, leucine, valine, and tryptophan, which were reversed by exercise treatment in both the plasma and skeletal muscle. Transcriptomic analysis of gene expression profiles in the skeletal muscle revealed several key pathways involved in the beneficial effects of exercise on metabolic homeostasis. In addition, integrative transcriptomic and metabolomic analyses uncovered strong correlations between the concentrations of bioactive metabolites and the expression levels of genes involved in energy metabolism, insulin sensitivity, and immune response in the skeletal muscle. This work established two models of exercise intervention in obese mice and provided mechanistic insights into the beneficial effects of exercise intervention on systemic energy homeostasis.

## Introduction

The prevalence of obesity and type 2 diabetes mellitus (T2DM) has been rapidly rising. Approximately 462 million people worldwide are affected by T2DM, among which >1 million die each year (Khan et al., 2020). Compared to healthy individuals, those with either obesity or T2DM show disruptions to their transcriptomic and metabolomic profiles. These are mainly characterized by alterations in the glucose, lipid, and/or amino acid metabolism (Varemo et al., 2015; Scott et al., 2016). Lifestyle management of patients is a crucial factor in preventing and managing obesity and T2DM, and exercise intervention is a central component in all obesity and T2DM prevention programs. Regular physical activity helps not only prevent the onset of T2DM, but can also improve T2DM-related variables such as body mass index, glycemic control and variability, insulin sensitivity, lipid profile, oxidative stress/antioxidative capacity, and/or chronic inflammation (Bennetsen et al., 2020; Esefeld et al., 2021; Meuffels et al., 2022). Despite such well-recognized benefits of exercise on metabolic homeostasis, the pleotropic effects of biomarkers and molecular transducers regulated by exercise remain poorly understood.

The skeletal muscle, one of the predominant sites of glucose disposal, plays a critical role in glycemic and metabolic homeostasis (DeFronzo and Tripathy, 2009). Physical activity provides benefits partly through extensive metabolic and molecular remodeling of the skeletal muscle in response to exercise (Egan and Zierath, 2013). For example, the skeletal muscle uniquely responds to exercise with increased sensitivity to subsequent insulin stimulation (Sylow et al., 2021). Exercise training also alters the DNA methylation of specific genes and pathways within the skeletal muscle in people with varying degrees of insulin sensitivity (Garcia et al., 2022). The skeletal muscle adapts to exercise through a variety of pathways, including muscle contraction/ATP biosynthesis coupling, and energy utilization upon activation of mechano- and other metabolic sensors (Egan and Zierath, 2013; Camera et al., 2016; Fan and Evans, 2017). Although considerable effort has been made to reveal the comprehensive changes stimulated by exercise (Pilegaard et al., 2000; Mahoney et al., 2005; Catoire et al., 2012), many regulators of the skeletal muscle remain undiscovered.

At present, the development of metabolic diseases such as obesity and T2DM are linked with the disruption of multiple interconnected ‘omic’ layers (such as those of the transcriptome, epigenome, and metabolome). These omic investigations help unravel the integrative physiology underlying such diseases. Some studies have identified the function of metabolic networks in the development of insulin resistance (Nogiec et al., 2015; Duft et al., 2020), and others have elucidated the global transcriptional response of human muscle to exercise (Yang et al., 2022). However, though often useful and insightful, most previous studies simply focused on single omic data of either genome, transcriptome, metabolome, or proteome, and thus were not able to comprehensively elucidate the integrative physiology of the complex diseases. To break through the limitation of single omic data, a multi-omic effort to discover the potential cross-talk between the transcriptome and metabolome on exercise-regulated pathways is urgently required.

Overall, the principal aim of this study was to assess and analyze transcriptional and metabolic networks regulated by exercise in high-fat diet (HFD)-induced obese mice. To this end, we established two models of exercise intervention in obese mice to provide mechanistic insight into the beneficial effects of exercise intervention on systemic energy homeostasis.

## Results

### Treadmill running mitigates HFD-induced systemic glucose homeostasis dysregulation

**Figure 1:**
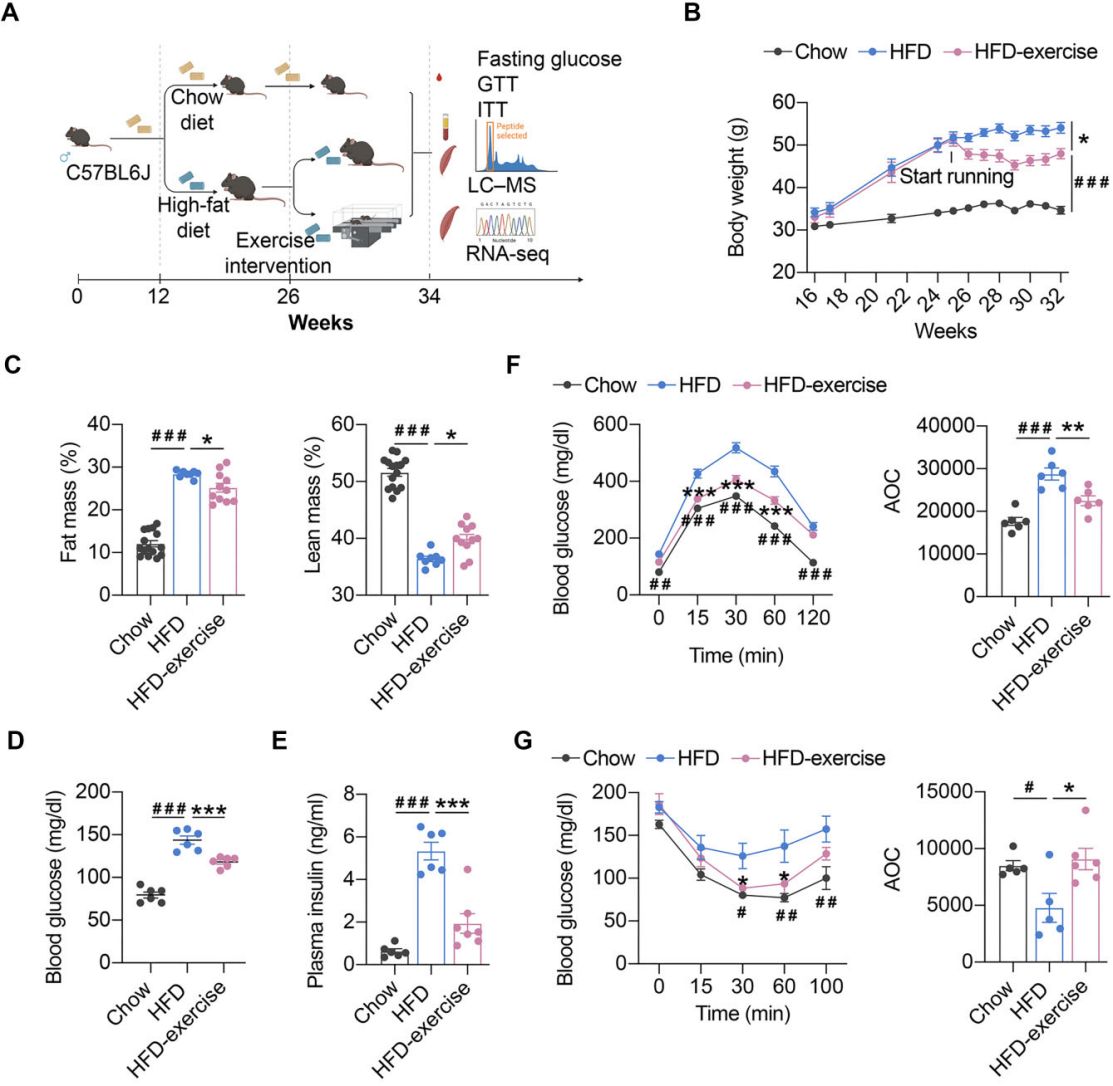
Exercise intervention mitigates HFD-induced systemic glucose homeostasis dysregulation. (**A**) Schematic representation of the experimental procedures. (**B**) Dynamic body weight changes in mice with indicated treatments (*n* = 6 mice per group). ^###^*P* < 0.001, HFD vs. Chow; *P < 0.05, HFD-exercise vs. HFD; two-way ANOVA. (**C**) Body composition of mice in the indicated groups (*n* = 15 mice for the Chow group, *n* = 8 mice for for the HFD group, *n* = 11 mice for the HFD-exercise group). ^###^*P* < 0.001, HFD vs. Chow; *P < 0.05, HFD-exercise vs. HFD; one-way ANOVA followed by Tukey's multiple comparisons test. (**D**) Overnight fasting blood glucose levels at 6 weeks post exercise intervention (*n* = 6 mice per group). ^###^*P* < 0.001, HFD vs. Chow; ***P < 0.001, HFD-exercise vs. HFD; one-way ANOVA followed by Tukey's multiple comparisons test. (**E**) Overnight fasting blood insulin levels at 8 weeks post exercise intervention (*n* = 6 mice for the Chow group, *n* = 6 mice for the HFD group, *n* = 7 mice for the HFD-exercise group). ^###^*P* < 0.001, HFD vs. Chow; ***P < 0.001, HFD-exercise vs. HFD; one-way ANOVA followed by Tukey's multiple comparisons test. (**F**) GTT at 8 weeks post exercise intervention (*n* = 6 mice per group) (left). ^##^*P* < 0.01, ^###^*P* < 0.001, HFD vs. Chow; ***P < 0.001, HFD-exercise vs. HFD; two-way ANOVA followed by Tukey's multiple comparisons test. AOC quantification of GTT results (right). ^###^*P* < 0.001, HFD vs. Chow; **P < 0.01, HFD-exercise vs. HFD; one-way ANOVA followed by Tukey's multiple comparisons test. (**G**) ITT at 7 weeks post exercise intervention (*n* = 5 mice for the Chow group, *n* = 5 mice for for the HFD group, *n* = 6 mice for the HFD-exercise group) (left). ^#^*P* < 0.05, ^##^*P* < 0.01, HFD vs. Chow; *P < 0.05, HFD-exercise vs. HFD; two-way ANOVA followed by Tukey's multiple comparisons test. AOC quantification of ITT results (right). ^#^*P* < 0.05, HFD vs. Chow; *P < 0.05, HFD-exercise vs. HFD; one-way ANOVA followed by Tukey's multiple comparisons test.

As shown in Figure 1, a murine prediabetic model was developed using HFD feeding. Exercise intervention (treadmill running) was conducted after 16-week HFD feeding (HFD-exercise group). The mice continuously fed a normal chow diet (Chow group) or HFD (HFD group) without exercise intervention were set up as control groups (Figure 1A). After another 8 weeks with or without exercise intervention, metabolic measurements including fasting glucose, glucose tolerance test (GTT), and insulin tolerance test (ITT), integrated multi-omics analysis (including metabolomics analysis in the plasma and skeletal muscle), and RNA sequencing (RNA-seq) analysis were performed for all groups (Figure 1A). As expected, HFD-fed mice showed sustained body weight gains compared to the Chow group, while exercise intervention significantly lessened the extent of the weight gain for the HFD-exercise group (Figure 1B). Exercise intervention also led to a reduction in the accumulation of fat mass and a reversal of lean mass loss induced by HFD (Figure 1C). Consistent with previous reports (Lang et al., 2019), HFD led to hyperglycemia, hyperinsulinemia, and impairments in both glucose tolerance and insulin sensitivity compared to the Chow group (Figure 1D–G). Exercise intervention was able to mitigate HFD-induced systemic glucose homeostasis dysregulation and improve blood glucose control. Compared to the HFD group, the HFD-exercise group showed decreased fasting glucose and plasma insulin levels, as well as improved glucose tolerance and insulin sensitivity (Figure 1D–G). Further investigation showed that the insulin sensitivity of local muscle, liver, and adipose tissue was increased after exercise intervention (Supplementary Figure S1). These results demonstrated that exercise intervention at the prediabetic stage can partially restore glucose homeostasis for diabetes remission in mice.

### Exercise training is linked to the altered plasma metabolism in HFD mice

**Figure 2:**
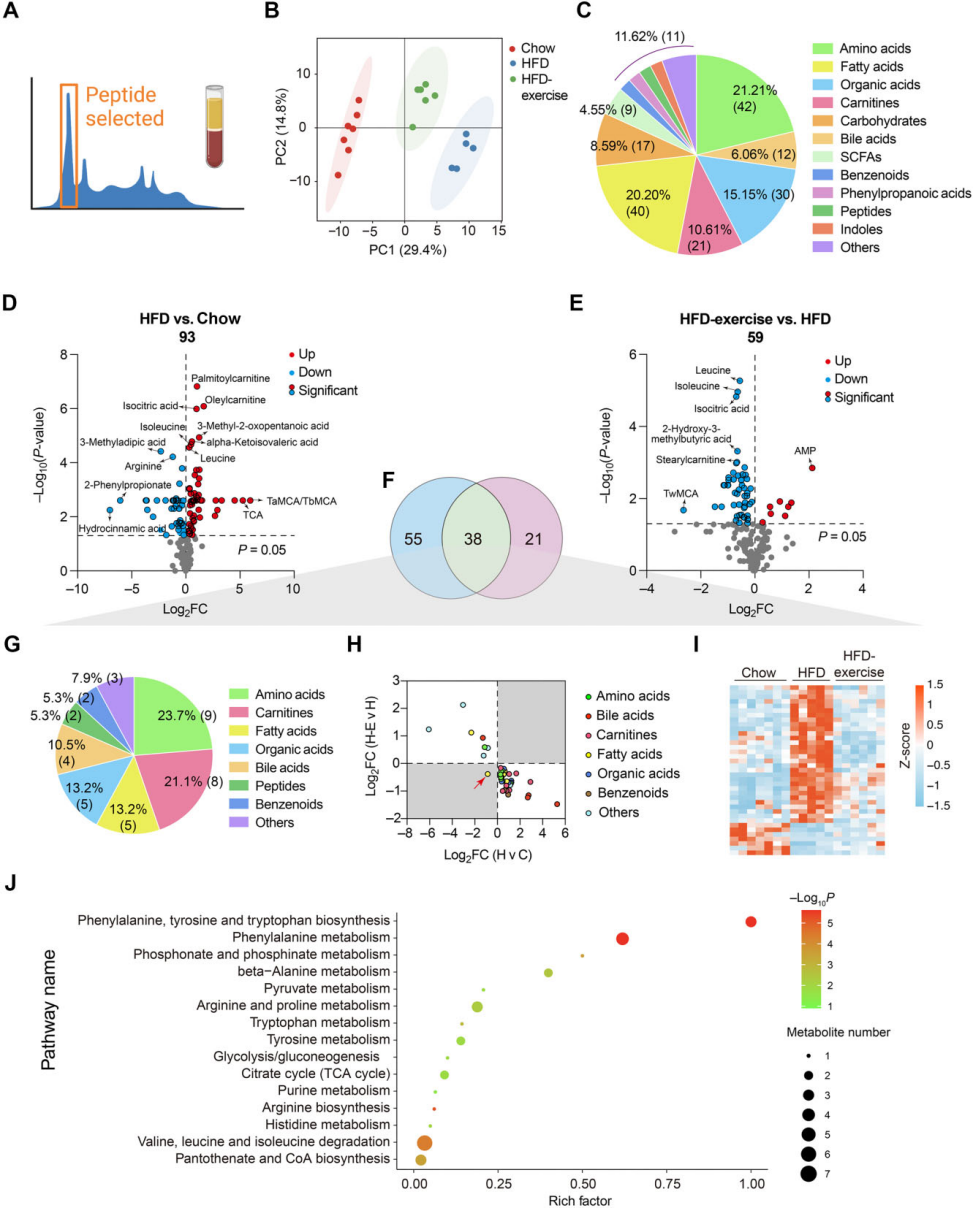
Treadmill running reverses HFD-induced changes in plasma metabolites. (**A**) Metabolomic data of plasma samples from the treadmill running experiment. (**B**) Scatter plot of PCA for metabolites of mice in the indicated groups. (**C**) Metabolite composition of plasma samples from indicated groups. SCFA, short-chain fatty acids. (**D**) Volcano plot of OPLS-DA model for each metabolite in HFD vs. Chow of plasma samples. The metabolites with log_10_(*P-*value) > 4 or |log_2_FC| ≥ 5 were labelled. (**E**) Volcano plot of OPLS-DA model for each metabolite in HFD-exercise vs. HFD of plasma samples. The metabolites with log_10_(*P-*value) > 3 or |log_2_FC| ≥ 2 were labelled. (**F**) Venn plot showing the overlap of differential metabolites shown in **D** and **E**. (**G**) Composition of overlapped metabolites (green shaded in **F**) in the Venn plot. (**H**) Scatter plot demonstrating that the changes of overlapped metabolites in the plasma were induced by HFD and mitigated by exercise. H v C, HFD vs. Chow; H-E v H, HFD-exercise vs. HFD. (**I**) Relative content heatmap for overlapped metabolites of mice in the indicated groups (*n* = 7 mice for the Chow group, *n* = 5 mice for the HFD group, *n* = 6 mice for the HFD-exercise group). Scale bar shows Z-score. (**J**) Bubble plot of significantly altered pathways (*P* < 0.05) reversed by exercise intervention. The horizontal coordinate is the extent to which the pathway is affected. The number of differential metabolites in the pathway is represented by graphs of different sizes. The *P*-values calculated by the enrichment analysis are described in terms of their color intensity.

To globally evaluate exercise-induced circulating metabolites, targeted metabolomics was performed with blood plasma samples from HFD-exercise mice following long-term exercise of treadmill running and the control groups (Figure 2A). Notably, principal component analysis (PCA) of the metabonomics data showed a clear separation among the three groups. The pattern of plasma metabolites of the Chow group more resembled to that of the HFD-exercise group rather than the HFD group, suggesting that the HFD-altered plasma metabolites might have been partly reversed after exercise intervention (Figure 2B). Among all 198 detectable plasma metabolites from each group, amino acids (21.21%), fatty acids (20.20%), and organic acids (15.15%) were identified to have top-ranked proportions (Figure 2C). Volcano plots of an OPLS-DA model together with univariate statistics displayed variable contribution (variable importance in the projection, VIP), variable reliability (correlation coefficients, Corr.Coeffs), fold change (FC), and *P-*value for each metabolite (Figure 2D and E; Supplementary Figure S2). We then analyzed all the annotated metabolites in the plasma and found 93 differential metabolites between the HFD and Chow groups and 59 differential metabolites between the HFD-exercise and HFD groups (VIP > 1, *P* < 0.05). Among these differential metabolites, 38 overlapped (Figure 2F and Table 1), in which amino acids, carnitines, and fatty acids composed 23.7%, 21.4%, and 13.2%, respectively (Figure 2G). Further investigation demonstrated that 37 of the overlapped metabolites were induced by HFD and reversed by exercise training, leaving only one metabolite not showing such reversal (Figure 2H and I). The Kyoto Encyclopedia of Genes and Genomes (KEGG) pathway enrichment analysis revealed that these 37 reversed metabolites could be mapped onto 27 metabolic pathways. By removing the pathways with an impact value of 0, 15 metabolic pathways were established, in which 10 belonged to the amino acid metabolism and 3 were metabolically related to glucose (Figure 2J). These results suggested that exercise intervention can reverse some of the overnutrition-induced changes in plasma metabolite levels.

**Table 1:** The overlapped metabolites in the plasma of the two compared groups.

		HFD vs. Chow			HFD-exercise vs. HFD		
Class	Metabolite	Ratio	*P*-value	VIP	Ratio	*P*-value	VIP
Amino acids	beta-Alanine	0.4557	0.0025	1.3250	1.5075	0.0271	1.2331
	Isoleucine	1.3932	0.0000	1.5600	0.6426	0.0000	1.7905
	Leucine	1.2506	0.0000	1.4852	0.6804	0.0000	1.8272
	Ornithine	2.2846	0.0016	1.4743	0.6012	0.0071	1.5746
	Phenylalanine	1.3051	0.0076	1.3500	0.7077	0.0031	1.7187
	Proline	1.2069	0.0329	1.0047	0.7594	0.0052	1.5130
	Tryptophan	1.2612	0.0010	1.3258	0.8199	0.0029	1.5238
	Tyrosine	1.3757	0.0014	1.4145	0.8336	0.0382	1.1529
	Valine	1.2446	0.0191	1.1402	0.7070	0.0031	1.6843
Carnitines	2-Methylbutyroylcarnitine	1.7471	0.0025	1.3540	0.5044	0.0043	1.6365
	Butyrylcarnitine	2.0816	0.0021	1.4674	0.5108	0.0027	1.7050
	Dodecanoylcarnitine	1.2252	0.0420	1.1584	0.8962	0.0054	1.5206
	Hexanylcarnitine	1.7309	0.0013	1.3581	0.5757	0.0020	1.5720
	Isovalerylcarnitine	1.3826	0.0225	1.1726	0.5000	0.0043	1.7284
	Oleylcarnitine	3.1747	0.0000	1.6036	0.7809	0.0132	1.3646
	Palmitoylcarnitine	2.0608	0.0000	1.5910	0.7685	0.0014	1.6036
	Stearylcarnitine	7.5675	0.0057	1.6257	0.6301	0.0010	1.6300
Fatty acids	2-Hydroxy-3-methylbutyric acid	1.7840	0.0003	1.5583	0.6408	0.0005	1.7646
	2-Methy-4-pentenoic acid	1.4786	0.0029	1.2252	0.7610	0.0186	1.3151
	3-Methyladipic acid	0.2011	0.0000	1.4802	2.1771	0.0310	1.2252
	DHA	0.5514	0.0177	1.2671	0.7666	0.0345	1.3044
	Octanoic acid	1.5618	0.0303	1.1514	0.8302	0.0494	1.2700
Organic acids	3-Methyl-2-oxopentanoic acid	2.3539	0.0000	1.5536	0.6199	0.0011	1.6243
	alpha-Ketoisovaleric acid	1.4918	0.0000	1.5131	0.8751	0.0147	1.3524
	Isocitric acid	1.9926	0.0000	1.5718	0.6202	0.0000	1.8155
	Ketoleucine	2.3768	0.0002	1.5533	0.6502	0.0023	1.6459
	Pyruvic acid	1.2966	0.0433	1.0486	0.6369	0.0076	1.6239
Benzenoids	Ortho-hydroxyphenylacetic acid	1.5429	0.0117	1.2708	0.5099	0.0031	1.5472
	Phenylpyruvic acid	1.9453	0.0025	1.4361	0.4580	0.0043	1.7056
Bile acids	NorDCA	0.4082	0.0228	1.0544	1.9048	0.0123	1.3438
	TCA	38.9488	0.0025	1.2354	0.3591	0.0173	1.2352
	TCDCA	6.8307	0.0025	1.4305	0.4490	0.0043	1.5239
	TDCA	6.4974	0.0092	1.1176	0.4248	0.0173	1.0460
Indoles	Indole-3-pyruvic acid	2.2016	0.0006	1.4607	0.5793	0.0030	1.6107
Nucleotides	AMP	0.1214	0.0101	1.0776	4.3713	0.0014	1.6108
Peptides	Anserine	0.5577	0.0025	1.4386	1.4815	0.0173	1.0606
	Glycylproline	0.4289	0.0025	1.3857	1.2188	0.0469	1.1136
Phenylpropanoic acids	2-Phenylpropionate	0.0148	0.0025	1.2195	2.3611	0.0173	1.1474

### Exercise training reverses metabolomic changes in HFD-fed mice

**Figure 3:**
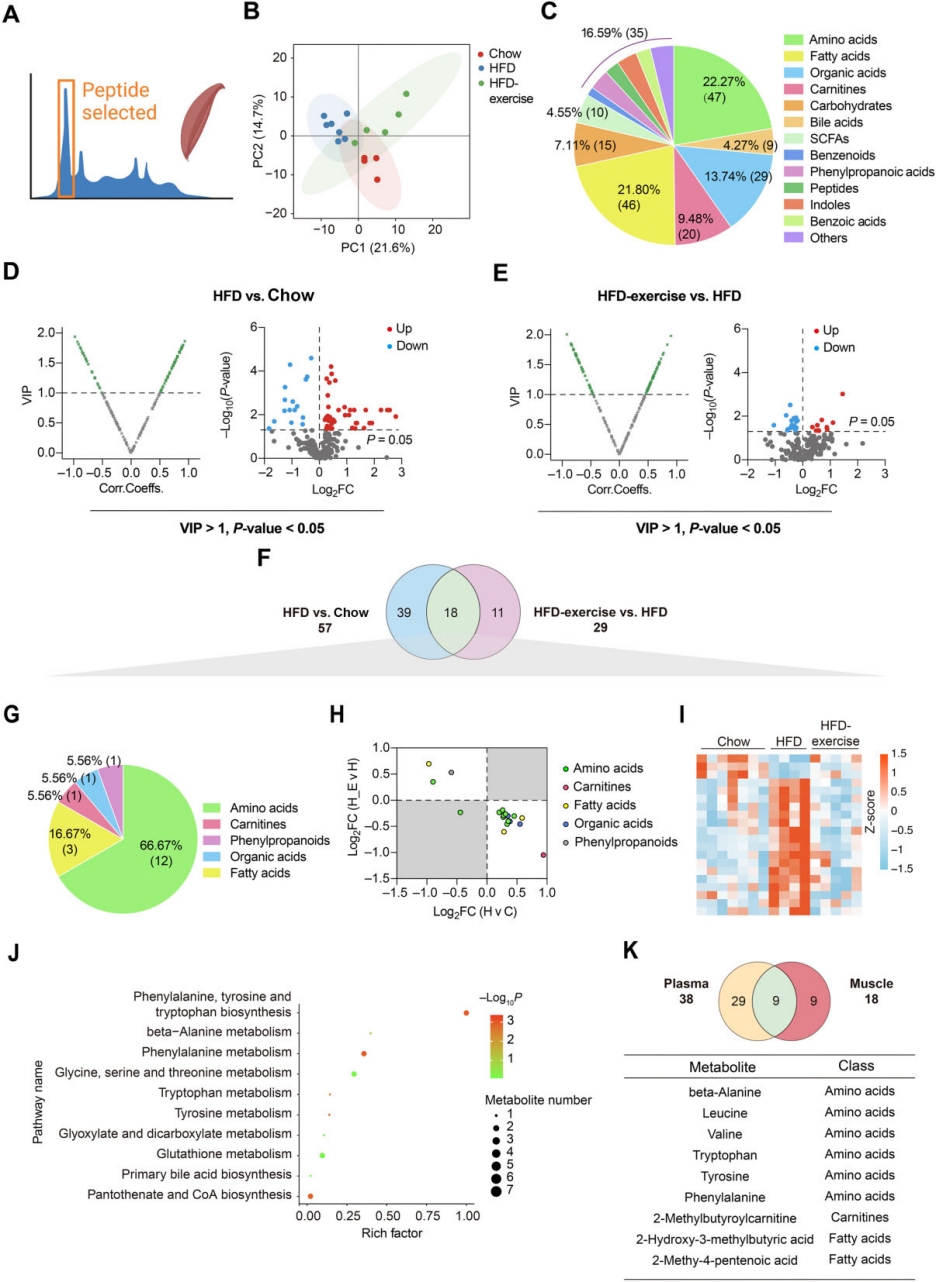
Treadmill running reverses HFD-induced changes of muscle metabolites. (**A**) Metabolomics of muscle samples from the treadmill running experiment. (**B**) Scatter plot of PCA for metabolites of mice in the indicated groups. (**C**) Metabolite composition of skeletal muscle samples from indicated groups. (**D** and **E**) Volcano plot of OPLS-DA model and univariate statistics for each metabolite in HFD vs. Chow (**D**) and HFD-exercise vs. HFD (**E**) of muscle tissues. (**F**) Venn plot showing the overlap of differential metabolites shown in **D** and **E**. (**G**) Composition of overlapped metabolites (green shaded in **F**) in the Venn plot. (**H**) Scatter plot demonstrating that the changes of overlapped metabolites in the skeletal muscle were induced by HFD and mitigated by exercise. H v C, HFD vs. Chow; H-E v H, HFD-exercise vs. HFD. (**I**) Relative content heatmap for overlapped metabolites of mice in the indicated groups (*n* = 7 mice for the Chow group, *n* = 4 mice for the HFD group, *n* = 5 mice for the HFD-exercise group). Scale bar shows Z-score. (**J**) Bubble plot of significantly altered pathways (*P* < 0.05) reversed by exercise intervention. The horizontal coordinate is the extent to which the pathway is affected, and the number of differential metabolites in the pathway is represented by graphs of different sizes. The *P*-values calculated by the enrichment analysis are described in terms of their color intensity. (**K**) Venn plot showing the overlap of exercise reversed metabolites in plasma and skeletal muscle samples. Table showing the overlapped metabolites (green shaded in the Venn plot) of plasma and skeletal muscle samples.

Targeted metabolomics analysis of quadriceps (Quad) was also performed to reveal changes of metabolites in the skeletal muscle (Figure 3A). Consistently, the metabolic profile of the Chow group shared more similarities with that of the HFD-exercise group rather than the HFD group (Figure 3B). A total of 211 metabolites from the skeletal muscle were identified, made up of amino acids (22.27%), fatty acids (21.80%), and organic acids (13.74%) (Figure 3C). According to VIP > 1 and *P-*value < 0.05, 57 and 29 differential metabolites were identified between the HFD and Chow groups and between the HFD-exercise and HFD groups, respectively (Figure 3D and E). Further analysis found that 18 of these metabolites overlapped (Figure 3F and Table 2), which mainly belonged to the classes of amino acids (66.67%) and fatty acids (16.67%) (Figure 3G). In addition, 17 of the overlapped metabolites showed reversibility of the overnutrition-induced changes after exercise intervention, among which 14 were increased by HFD feeding and relieved by exercise training (Figure 3H and I). The skeletal muscle metabolite profiles of HFD-exercise mice were similar to that of Chow mice in the heatmap (Figure 3I). KEGG pathway enrichment analysis showed that these differential metabolites were enriched in various metabolic pathways, especially those related to the amino acid metabolism (Figure 3J).

**Table 2:** The overlapped metabolites in the muscle of the two compared groups.

		HFD vs. Chow			HFD-exercise vs. HFD		
Class	Metabolite	Ratio	*P*-value	VIP	Ratio	*P*-value	VIP
Amino acids	Alanine	1.1533	0.0171	1.6026	0.8518	0.0176	1.6697
	beta-Alanine	0.5367	0.0061	1.5661	1.2777	0.0317	1.5396
	Glycine	0.7366	0.0002	1.6926	0.8518	0.0332	1.3884
	Homoserine	1.2609	0.0044	1.4630	0.7300	0.0030	1.8400
	Leucine	1.3701	0.0001	1.7618	0.8106	0.0266	1.6013
	Phenylalanine	1.3058	0.0129	1.4542	0.7560	0.0159	2.0091
	Pyroglutamic acid	1.2726	0.0188	1.4266	0.8131	0.0346	1.7513
	Serine	1.1995	0.0424	1.4227	0.8775	0.0159	1.6547
	Threonine	1.2072	0.0002	1.7390	0.7999	0.0125	1.6793
	Tryptophan	1.2127	0.0403	1.3491	0.8172	0.0358	1.6597
	Tyrosine	1.2402	0.0013	1.7700	0.8323	0.0447	1.4339
	Valine	1.2801	0.0350	1.3997	0.7573	0.0259	1.7663
Fatty acids	2-Hydroxy-3-methylbutyric acid	1.2167	0.0335	1.2733	0.6580	0.0091	1.6919
	2-Methy-4-pentenoic acid	1.5013	0.0003	1.6816	0.7903	0.0274	1.5599
	Undecanoic acid	0.5110	0.0025	1.5774	1.6217	0.0148	1.6674
Carnitines	2-Methylbutyroylcarnitine	1.9188	0.0106	1.6736	0.4839	0.0257	1.8539
Organic acids	Lactic acid	1.4636	0.0206	1.6157	0.7300	0.0347	1.6410
Phenylpropanoids	3,4-Dihydroxyhydrocinnamic acid	0.6632	0.0134	1.5231	1.4462	0.0255	1.6963

When comparing the differential metabolites in the plasma (38 metabolites) and muscle (18 metabolites) samples, the same variation trend was observed in nine metabolites, beta-alanine, leucine, valine, tryptophan, tyrosine, phenylalanine, 2-methylbutyroylcarnitine, 2-hydroxy-3-methylbutyric acid, and 2-methy-4-pentenoic acid (Figure 3K). These results suggested that exercise intervention has a great influence on the muscle and plasma metabolism in obese mice, primarily through altering the amino acid metabolism.

### Exercise training reverses the skeletal muscle transcriptome in HFD-fed mice

**Figure 4:**
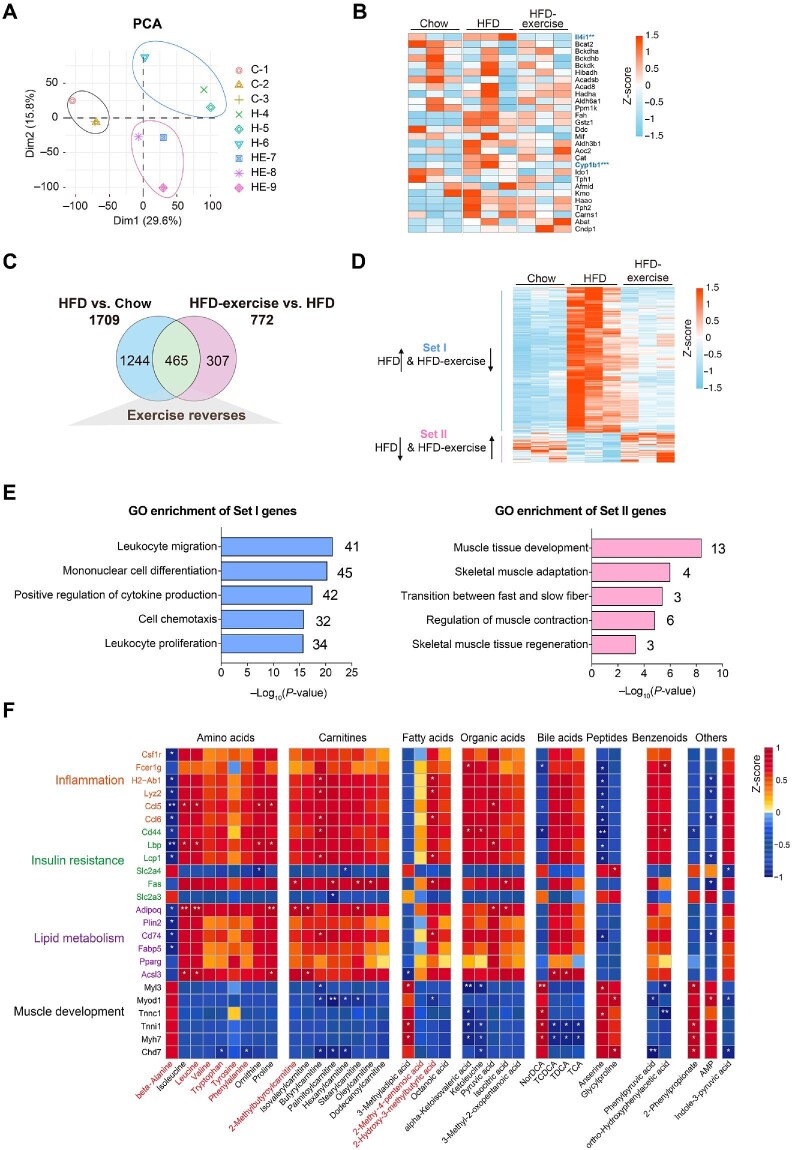
Exercise training reverses the skeletal muscle transcriptome in HFD-fed mice. (**A**) Scatter plot of PCA for genes of mice in the indicated groups. (**B**) Heatmap of metabolite-related genes for mice in the indicated groups (*n* = 3 mice per group). Scale bar shows Z-score. **P < 0.01 and ***P < 0.001. (**C**) Venn plot showing the number of genes with expression induced by HFD and/or reversed by HFD-exercise (|log_2_FC| > 0.3, *P* < 0.05). (**D**) Heatmap of reversed genes for mice in the indicated groups (*n* = 3 mice per group). Scale bar shows Z-score. Set I: genes with expression increased in the HFD group and decreased in the HFD-exercise group; Set II: genes with expression decreased in the HFD group and increased in the HFD-exercise group. (**E**) GO analysis of genes in Set I and Set II. (**F**) Heatmap of the Spearman's correlation coefficients between changes in reversed genes and metabolite alterations caused by exercise intervention. *P < 0.05 and **P < 0.01.

To investigate whether the metabolic benefits coincide with the transcriptional signature, RNA-seq analysis was performed to examine the gene expression profiles in Quad muscles from the Chow, HFD, and HFD-exercise groups. Clear separation was found among the three groups via PCA (Figure 4A). Then, the RNA-seq data of genes related to the catabolic pathways were analyzed. Unexpectedly, the results showed that the expression levels of these related genes were not changed significantly in the HFD-exercise group compared to the HFD group, suggesting that exercise intervention regulated the amino acid changes in the muscle not through altering the catabolic pathway of the muscle itself (Figure 4B). Differential transcript analysis showed that a total of 1709 genes in Quad muscles expressed differentially after HFD feeding, while 772 genes expressed differentially after exercise intervention (Figure 4C). HFD feeding significantly upregulated the expression of 1296 genes in the skeletal muscle, and 386 of them were then significantly downregulated in response to exercise training. These 386 genes were classified into Set I. Among the 413 genes downregulated by HFD feeding, 79 genes were subsequently upregulated by exercise intervention and were classified into Set II (Figure 4C and D). Gene Ontology (GO) genetic pathway analysis on the 465 differential transcripts indicated that genes in Set I were mainly involved in the inflammation-related pathways, while genes in Set II were enriched in the areas of muscle development and function (Figure 4E). These findings suggested that exercise training may be effective in reversing HFD-induced loss of muscle tissues by regulating muscle contraction and development and reversing HFD-induced immune system disorders.

### Integrated analysis reveals that exercise training is associated with insulin resistance pathways

According to the generally accepted associations of typical inflammation, insulin resistance, lipid metabolism, and muscle development pathways with the differential metabolisms, we discovered seven main metabolic categories that showed links to the expression of genes related to these pathways (Figure 4F). Most of amino acids, carnitines, fatty acids, organic acids, bile acids, and benzenoids were positively associated with inflammation, insulin resistance, and lipid metabolism pathways, whereas negatively associated with muscle morphogenesis pathways. The heatmap (Figure 4F) showed that exercise-induced upregulation of metabolites positively correlated with the induction of several inflammation-related genes (|*r*| > 0.7, *P* < 0.05) and fatty acid biosynthetic genes (|*r*| > 0.7, *P* < 0.05). To investigate whether macrophages were altered by exercise training, we analyzed the gene expression of M1-like and M2-like macrophage markers in the quadriceps from the Chow, HFD, and HFD-exercise groups, and found that almost all the measured genes, except Arg1, were upregulated in the HFD group and then downregulated after exercise intervention (Supplementary Figure S3).

Exercise-induced reductions in muscle amino acids, carnitines, fatty acids, and organic acids correlated with the upregulation of a similar set of genes involved in insulin resistance as well as the genes involved in muscle development (Figure 4F). Among these metabolites, beta-alanine, isoleucine, leucine, valine, tryptophan, tyrosine, 2-methylbutyroylcarnitine, isovalerylcarnitine, 2-methy-4-pentenoic acid, and 2-hydroxy-3-methylbutyric acid in plasma samples were regulated in the same trends as in muscle samples. In addition, isoleucine and leucine are not only essential amino acids but also belong to branched-chain amino acids (BCAAs) essential for the attainment of muscle mass and strength. This result demonstrated a lower BCAA level in the skeletal muscle of the HFD-exercise group compared with the HFD group. The transcriptomic data of the skeletal muscle showed that muscle contraction and development pathways were upregulated in HFD-exercise mice compared with HFD mice (Figure 4D and E), suggesting that exercise intervention might reverse HFD-induced elevation of BCAA levels by utilizing these amino acids as the substrate for protein synthesis and muscle growth. To further clarify the relationship between metabolites and the transcriptome in the muscle, we analyzed the expression of genes that take part in the catabolic process of metabolites in the muscle (Supplementary Figure S4). The results showed that, consistent with the alteration of the metabolites, the expression of the related genes (Il4i1, Cyp1b1, Tph2, Haao, Fah, and Gstz1) was upregulated by HFD feeding and then downregulated after exercise intervention. These results further suggested that exercise-induced alterations in metabolites contribute to the improvement of glucose metabolism in mice after exercise intervention.

### Wheel running intervention also mitigates HFD-induced systemic glucose homeostasis dysregulation

**Figure 5:**
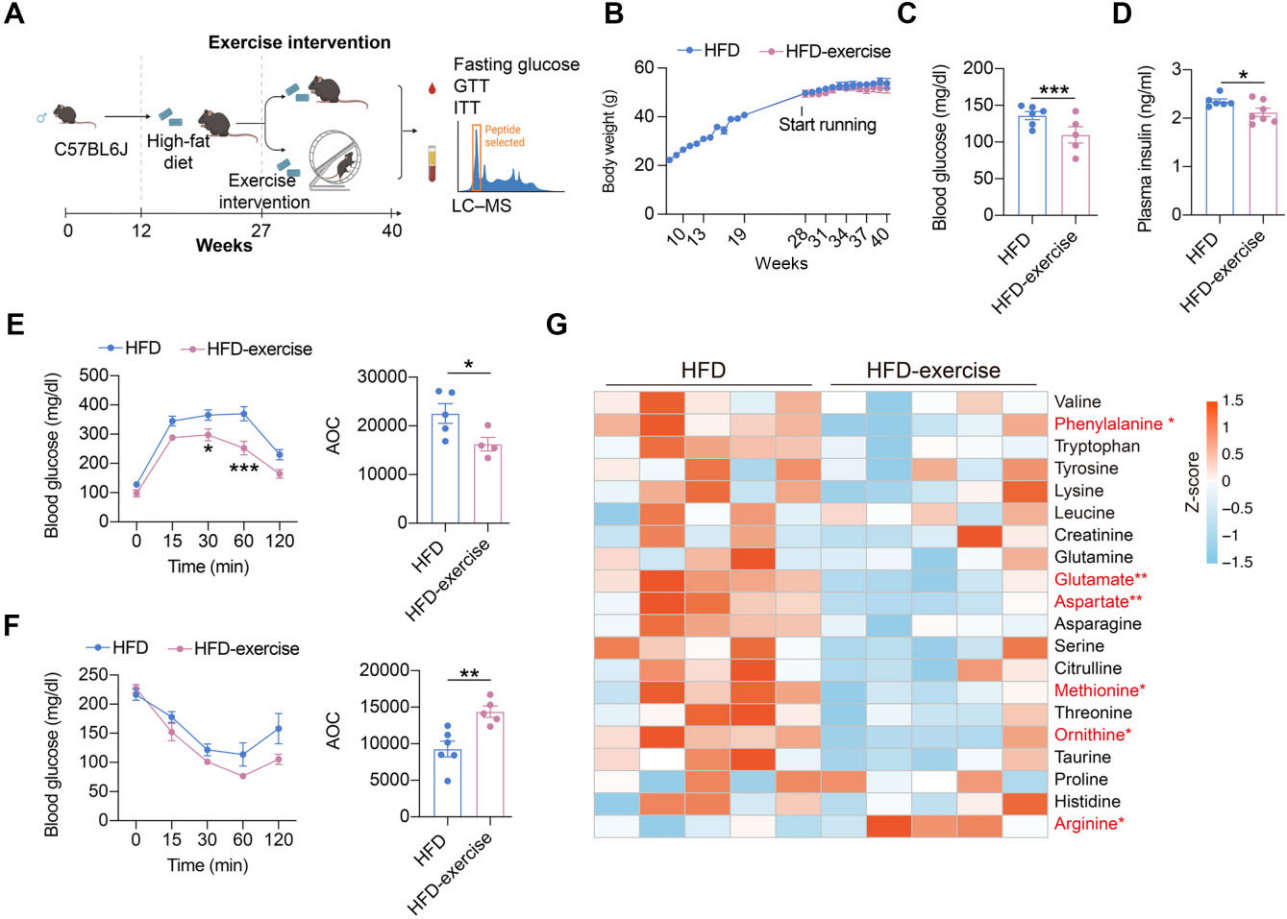
Wheel running can also alter glucose metabolism and metabolite levels. (**A**) Schematic representation of the experimental procedures. (**B**) Dynamic body weight changes in mice with indicated treatments (*n* = 11 mice for the HFD group, *n* = 9 mice for the HFD-exercise group). (**C**) Overnight fasting blood glucose levels at 7 weeks post exercise intervention (*n* = 6 mice for the HFD group, *n* = 5 mice for the HFD-exercise group). ***P < 0.001, two-tailed unpaired Student's *t*-test. (**D**) Overnight fasting plasma insulin levels at 7 weeks post exercise intervention (*n* = 6 mice for the HFD group, *n* = 7 mice for the HFD-exercise group). *P < 0.05, two-tailed unpaired Student's *t*-test. (**E**) GTT at 8 weeks post exercise intervention (*n* = 5 mice for the HFD group, *n* = 4 mice for the HFD-exercise group) (left). *P < 0.05, ***P < 0.001, two-way ANOVA followed by Sidak's multiple comparisons test. AOC quantification of GTT results (right). *P < 0.05, two-tailed unpaired Student's *t*-test. (**F**) ITT at 9 weeks post exercise intervention (*n* = 6 mice for the HFD group, *n* = 5 mice for the HFD-exercise group) (left). Two-way ANOVA followed by Sidak's multiple comparisons test. AOC quantification of ITT results (right). **P < 0.01, two-tailed unpaired Student's *t*-test. (**G**) Heatmap of relative abundance of amino acid metabolites in HFD and HFD-exercise mice (*n* = 5 mice per group). Scale bar shows Z-score. *P < 0.05, **P < 0.01, two-tailed unpaired Student's *t*-test.

In addition to the treadmill exercise, another form of exercise, wheel running, was also applied to examine the effect of exercise intervention on whole-body glucose metabolism and metabolites. We established a wheel running exercise mouse model, in which mice from the HFD-exercise group had free access to the running wheel in the cage, while mice from the HFD group lacked such access. Metabolic measurements, including fasting glucose, GTT, and ITT, were performed at the end of the intervention, with concurrent measurement of the amino acid content of the plasma (Figure 5A). Differing from treadmill running, wheel running did not lead to a significant reduction in body weight gain (Figure 5B). However, decreased fasting glucose and insulin levels and improved glucose tolerance and insulin sensitivity after wheel running were observed, in consistent with that by treadmill running intervention (Figure 5C–F). These results demonstrated that wheel running intervention could also improve systemic glucose homeostasis in mice with overnutrition.

Since the treadmill training model suggested that amino acid metabolism pathways play a key role in improving glucose metabolism in HFD mice, we then compared amino acid levels in the plasma of HFD and HFD-wheel running mice. The results showed a significant reduction in phenylalanine, glutamate, aspartate, methionine, and ornithine, as well as a decreasing trend in valine, tryptophan, tyrosine, lysine, leucine, creatinine, glutamine, asparagine, serine, citrulline, threonine, taurine, proline, and histidine, after wheel running intervention (Figure 5G). These results suggested that wheel running might also improve systemic glucose metabolism by altering the amino acid metabolism.

## Discussion

Exercise training has gained increasing attention for its ability to counteract obesity and diabetes. In the present study, we used several unbiased approaches to assess the impact of exercise on the metabolomic and transcriptomic profiling of the plasma and skeletal muscle in HFD-fed mice. First, as previously recognized, we confirmed that exercise training indeed reversed HFD-induced obesity and impairments in glucose tolerance. We then went deeper to investigate several amino acids, such as beta-alanine, leucine, valine, and tryptophan, which may mediate the effects of exercise on obese mice. We further looked into the specific gene expression patterns linked with the initial phase of the adaptive response in the skeletal muscle via comparisons between exercise and non-exercise groups. We were finally able to provide a new conceptual framework by which the amino acid metabolism may contribute to exercise-mediated benefits upon the systemic glucose metabolism.

Given that a number of reports have demonstrated that exercise training can improve HFD-induced obesity and mitigate impaired glucose tolerance (Cheng et al., 2022; Yang et al., 2022), one possible mechanism is that such exercise improves these dysfunctions by remodeling the skeletal muscle (Bassel and Olson, 2006). If so, this remodeling response would likely involve the activation of intracellular signaling pathways and consequent genetic reprogramming, leading to alterations in muscle mass, contractile properties, and metabolic states (Bassel and Olson, 2006). Correspondingly, previous studies have reported that the movement of the muscle fibers enhances transmembrane glucose transport via the increase of glucose transporter 4, which leads to blood glucose reduction under physical exertion (Hussey et al., 2012; Howlett et al., 2013; Richter and Hargreaves, 2013; Stanford and Goodyear, 2014). Other proposed mechanisms include stronger insulin binding to muscular insulin receptors, an increase in the number of muscular insulin receptors, increased activity of energy metabolism enzymes, and/or an increase in muscular capillary density (Stanford and Goodyear, 2014; Sylow et al., 2021).

Exercise is known to enhance the catabolism of amino acids, including beta-alanine, leucine, valine, tryptophan, tyrosine, and phenylalanine, in both the plasma and skeletal muscle. Among these amino acids, both leucine and valine are BCAAs. Consistent with previous studies (Gunther et al., 2020), our study demonstrated lower BCAA levels in both the plasma and skeletal muscle of the HFD-exercise group compared with the HFD group. It is reported that BCAAs, and their metabolic by-products, strongly associate with insulin resistance (Newgard et al., 2009). Exercise accelerates BCAA biosynthesis and degradation, and a tight regulation of BCAA catabolism in the muscle is important for the homeostasis of the muscle energy metabolism and adaptation to exercise training (Xu et al., 2017). The pathway leading to high levels of BCAAs in obesity is not completely understood but may involve chronic low-grade inflammation that prompts pro-inflammatory gene expression in the adipose tissue and determines further obesity impacts on metabolic health (Zhang et al., 2016; Mu et al., 2018). BCAAs have also been consistently identified as risk factors for cardiometabolic diseases due to their strong associations with insulin resistance (Yoon, 2016) and with evidence accumulating for their causal effects (Wang et al., 2011; Chen et al., 2016; Ke et al., 2019). Reduced BCAA concentrations achieved through lifestyle modifications over a prolonged period may therefore result in lower risks of T2DM (Kujala et al., 2016). However, discrepancies do appear in different studies related to this claim. For example, in one study, a bout of 12-week endurance and resistance-exercise training failed to result in any significant reduction in BCAAs levels, despite improvements in insulin sensitivity (Glynn et al., 2015). Previous studies have demonstrated that BCAA supplementation impaired the beneficial effect of exercise on glycolipid metabolism in obese mice (Zhang et al., 2022), and BCAA contributes to the development of obesity-associated insulin resistance in obese humans (Newgard et al., 2009). Meta-analysis showed that beta-alanine supplementation could increase muscle carnosine concentration and improve exercise capacity and performance (Saunders et al., 2017). Ueda et al. (2018) found that tryptophan significantly improved glucose tolerance in both lean and diet-induced obese mice, but the extent of improvement was bigger in the obese mice with augmented glucose-stimulated insulin secretion enhancement (Ueda et al., 2018). Besides, a human study suggested that ingestion of L-phenylalanine, but not D-phenylalanine, increased insulin, glucagon, and GIP concentrations whereas reduced postprandial glucose levels without any obvious effects on appetite (Amin et al., 2021).

Gene transcription is a key process controlling skeletal muscle phenotype and, consequently, metabolic health. Both pathological and physiological cues, as coupled with impaired and improved insulin sensitivity, respectively, link to the extensively remodeling of the skeletal muscle transcriptome (Choi et al., 2005; Keller et al., 2011; Varemo et al., 2015). In this study, exercise led to differential gene expression patterns in the skeletal muscle, including downregulation of immune-related gene categories, affecting leukocyte migration and mononuclear cell differentiation, and positive regulation of cytokine production, reflecting an anti-inflammatory effect. It is generally accepted that regular and moderate physical activity is inversely correlated with systemic low-grade inflammation, supporting the notion that exercise exerts a protective effect on patients with chronic diseases through its anti-inflammatory action (Mazur et al., 2017). Additionally, muscle cells are thought to have the capacity to produce several hundreds of secreted factors, which have been termed ‘myokines’ (Bortoluzzi et al., 2006; Yoon et al., 2009). Myokines are believed to incorporate protective functions, including those with anti-inflammatory effects and/or specific effects, on the adipose tissue (Pedersen and Febbraio, 2012; Schnyder and Handschin, 2015). Therefore, the regulation of the expression of inflammatory genes may directly influence myokine secretion and therefore lower the muscle and global inflammatory status. This may partly explain the reduction in inflammatory markers such as C-reactive protein following long-term exercise (Mattusch et al., 2000; Stewart et al., 2007). In this study, we clearly observed the upregulated expression of genes related to the processes of muscle reconstruction and adaptation. During exercise, the skeletal muscle utilizes both muscle glycogen stores and circulating plasma glucose as fuel sources (Thyfault and Bergouignan, 2020). Muscle contractions, even at low intensity and low volume (Bergouignan et al., 2016), can activate both oxidative and non-oxidative glucose disposal and glucose uptake via insulin-dependent and insulin-independent mechanisms (Thyfault, 2008), thus optimizing insulin actions on both glucose oxidation and storage.

In addition, we compared plasma amino acids between HFD and HFD-wheel running mice and found lower levels of valine, serine, phenylalanine, and tryptophan in the HFD-wheel running group. This result was broadly consistent with the treadmill experiment. We also demonstrated that, unlike the un-exercised HFD group, exercise resulted in a differential transcriptional response with GO terms associated with the regulation of cell immunity and skeletal muscle plasticity. Upon successfully implementing transcript–metabolite correlation analysis, we were able to further demonstrate the predominant effect of exercise on the amino acid metabolism, insulin resistance, fatty acid biosynthesis, and muscle morphogenesis.

In conclusion, in this study, metabolic and transcriptional profiling revealed that exercise training reverses HFD-induced changes in metabolism and transcription. Several amino acid metabolism pathways and muscle development pathways impacted by exercise training are implicated with important roles in the improvement of obesity and T2DM.

## Materials and methods

### Animal models

Wild-type mice on the C57BL/6 J background were obtained from GemPharmatech Co., Ltd (Nanjing, China). All animal studies were performed according to procedures approved by the University Committee on Use and Care of Animals at the Zhejiang University. Mice were housed in 12-h/12-h light/dark cycles at an ambient temperature of 23°C. All animal experiments used age-matched male mice.

To generate diet-induced obese mouse models, 12-week-old C57BL/6 J male mice were fed either a chow diet (10 kcal% fat, 70 kcal% carbohydrate, and 20 kcal% protein; Jiangsu Xietong Pharmaceutical Bio-engineering Co., Ltd, 1010088) or a HFD (60 kcal% fat, 20 kcal% carbohydrate, and 20 kcal% protein; Research Diets, D12492) for 16 weeks. For exercise intervention, mice fed with HFD for 16 weeks were subjected to treadmill running or wheel running for 8 weeks.

### Metabolic measurements

Body fat and lean mass were measured using an NMR analyzer (Niumag, QMN06-090H). Fasting blood glucose levels were determined using a Bayer Contour blood glucometer by tail-snip blood sampling after overnight fasting (∼16 h).

### GTT and ITT

GTT and ITT were performed as previously described (Meng et al., 2017). For GTT, mice were fasted overnight (∼16 h) and intraperitoneally (i.p.) injected with glucose saline solution (1.2 g/kg body weight, glucose concentrations adjusted accordingly to obtain an equal injection volume for each mouse). Blood glucose levels were measured by tail-snip blood sampling pre-injection and 15, 30, 60, and 120 min post-injection. For ITT, mice were fasted for 4 h and i.p. injected with insulin saline solution (1 unit/kg body weight, insulin concentrations adjusted accordingly to equalize injection volume for each mouse). Blood glucose levels were measured by tail-snip blood sampling pre-injection and 15, 30, 60, and 120 min post-injection.

### Metabolomic analysis

For treadmill running mice, plasma samples (50 μl) and muscle samples (50 mg) from the Chow, HFD, and HFD-exercise mice were subjected to identification and quantification using a Q300 Metabolite Array. Raw data files were analyzed by QuanMET (V2.0; Metabo-Profile) to identify and quantify metabolites.

For wheel running mice, plasma samples (50 μl) from HFD and HFD-exercise mice were subjected to identification and quantification using a Q300 Metabolite Array as previously described (Gu et al., 2015; Zhang et al., 2021).

### RNA-seq and bioinformatics

For RNA-seq analysis, total muscle RNA samples were sent for library preparation and sequencing by the BGI group (Wuhan, China). In brief, mRNAs were enriched from total RNA and fragmented. These were then used for reverse transcription and second-strand cDNA synthesis. The cDNAs were tailed with adenine and ligated with adaptors for polymerase chain reaction (PCR) amplification and sequencing. PCR-amplified cDNA libraries were subjected to paired-end sequencing on a BGISEQ-500 system. Data were processed following the standard BGI mRNA analysis pipeline. Expression levels of mRNA were computed as fragments per kilobase of transcript per million mapped reads for statistical analysis, as performed using the Deseq2 (V.1.20.0) package (Love et al., 2014). Pathway grouping and enrichment studies, as well as GO analysis, were performed using clusterProfiler (V3.12.0). Pathway visualization was conducted using pathview (V1.26.0) (Yu et al., 2012; Luo and Brouwer, 2013).

### Statistical analysis

Statistical analyses were carried out using GraphPad Prism 8. Statistical differences were evaluated using two-tailed unpaired Student's *t*-test for comparisons between two groups and using analysis of variance (ANOVA) followed by appropriate *post hoc* analysis for comparisons of more than two groups. For ITT and GTT, two-way ANOVA with multiple comparisons test was used. The area of the curve (AOC) was calculated by subtracting the starting glucose value from the value at each time point (Virtue and Vidal, 2021) for each mouse, and statistical difference between two groups was evaluated using two-tailed unpaired Student's *t*-test. A *P*-value < 0.05 (*P < 0.05, **P < 0.01, ***P < 0.001, ^#^*P* < 0.05, ^##^*P* < 0.01, and ^###^*P* < 0.001) was considered statistically significant. Statistical methods and corresponding *P*-values for data are included in the figure legends. No statistical method was used to predetermine the sample size. The experiments were not randomized, and the investigators were not blinded to allocation during the experiments and outcome assessment.

## Supplementary Material

mjad016_Supplemental_File
