# Supplementary material for: An integrative profiling of metabolome and transcriptome in the plasma and skeletal muscle following an exercise intervention in diet-induced obese mice
Source: J Mol Cell Biol. 2023 Mar 7;15(3):mjad016. doi: 10.1093/jmcb/mjad016 (PMC10576543; doi:10.1093/jmcb/mjad016)
Supplement: mjad016_Supplemental_File [file mjad016_supplemental_file.pdf]

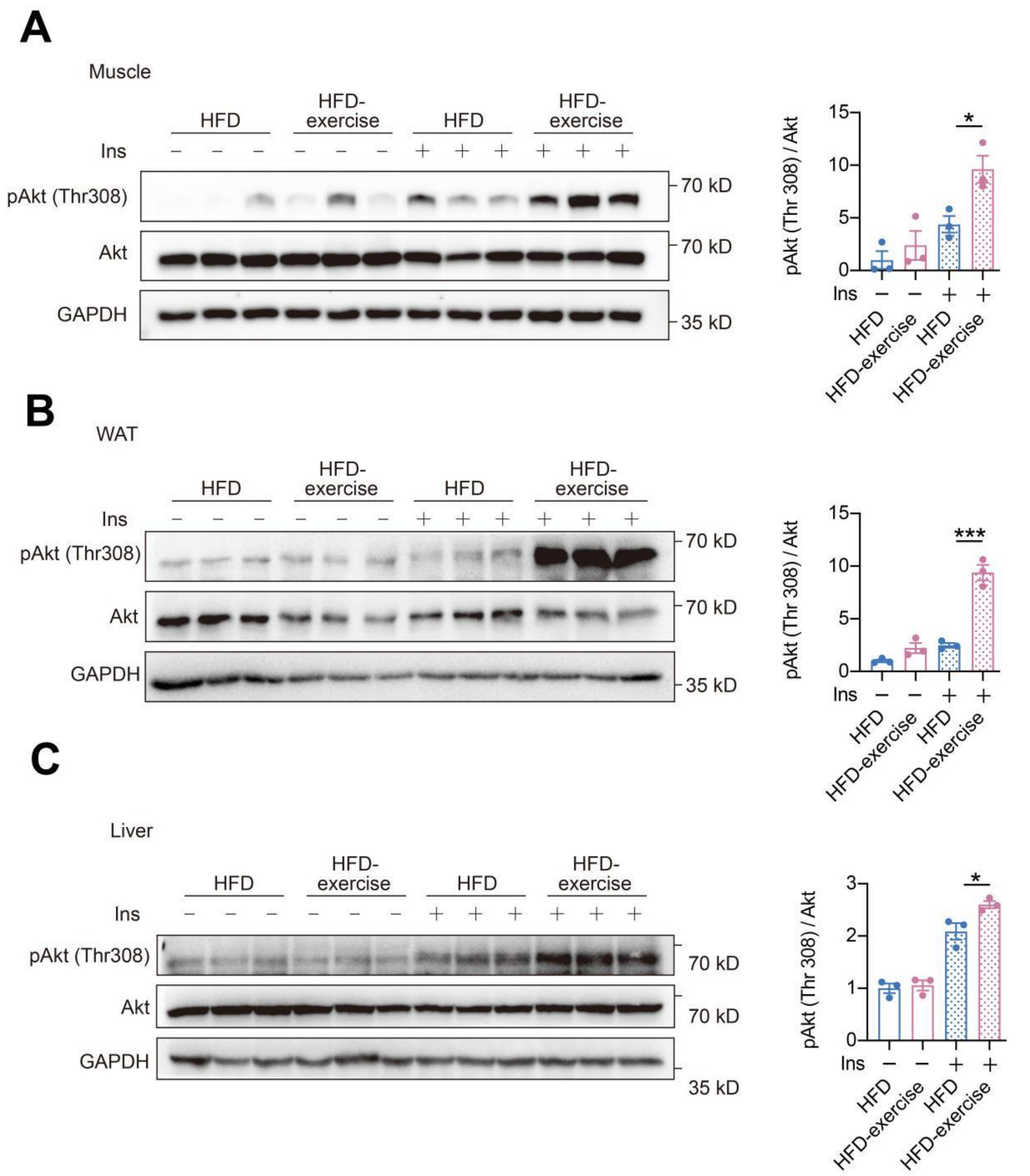

Supplementary Figure S1. Protein levels of major metabolic tissues from the indicated mice. Immunoblots of total protein lysates of the quadriceps muscle (**A**), adipose tissue (**B**), and liver (**C**) from HFD and HFD-exercise mice after intraperitoneally injected with saline or insulin (3.5 units/kg) for 10 min (left). Quantification of immunoblots (right). n = 3 mice per group. WAT, white adipose tissue; Ins, insulin. *P < 0.05, ***P < 0.001, two-tailed unpaired Student's t-test.

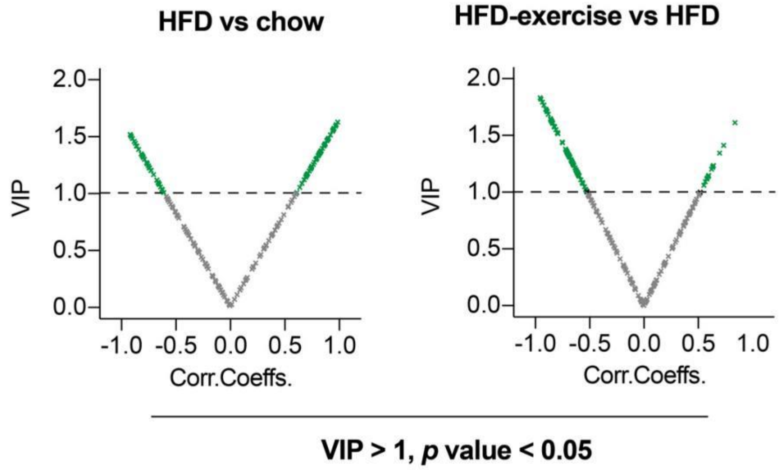

Supplementary Figure S2. Univariate statistics for each metabolite in plasma samples. Comparisons in HFD vs chow (left) and HFD-exercise vs HFD (right). VIP, variable importance in the projection; Corr.Coeffs, correlation coefficients.

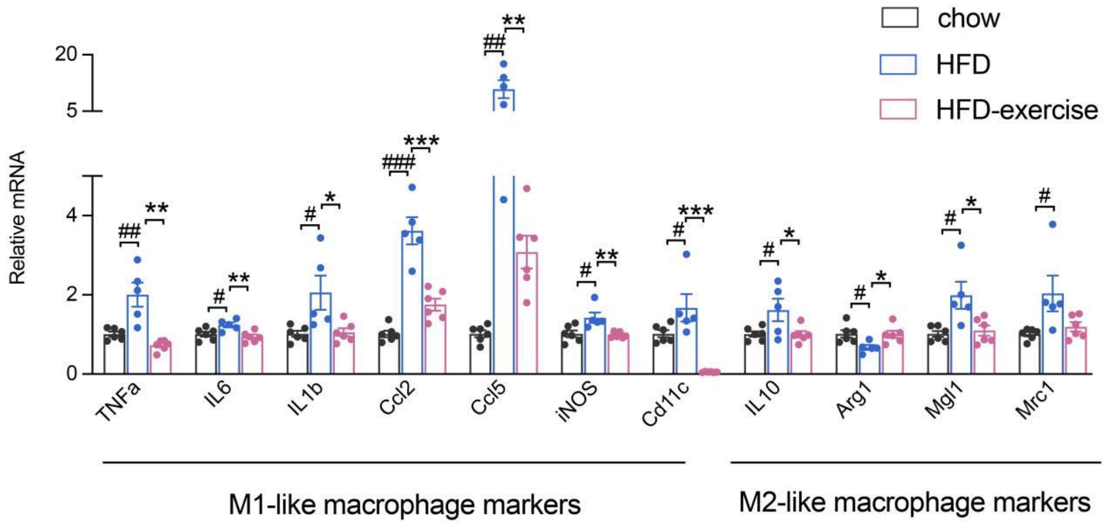

Supplementary Figure S3. Relative gene expression levels of M1-like and M2-like macrophage markers in skeletal muscles. Data represent mean ± SEM (n = 6 mice for the chow group, n = 5 mice for the HFD group, and n = 6 mice for the HFD-exercise group). #$P < 0.05$, ##$p < 0.01$, ###$p < 0.001$, HFD versus chow; *$P < 0.05$, **$P < 0.01$, ***$P < 0.001$, HFD-exercise versus HFD, one-way ANOVA followed by Tukey's multiple comparisons test.

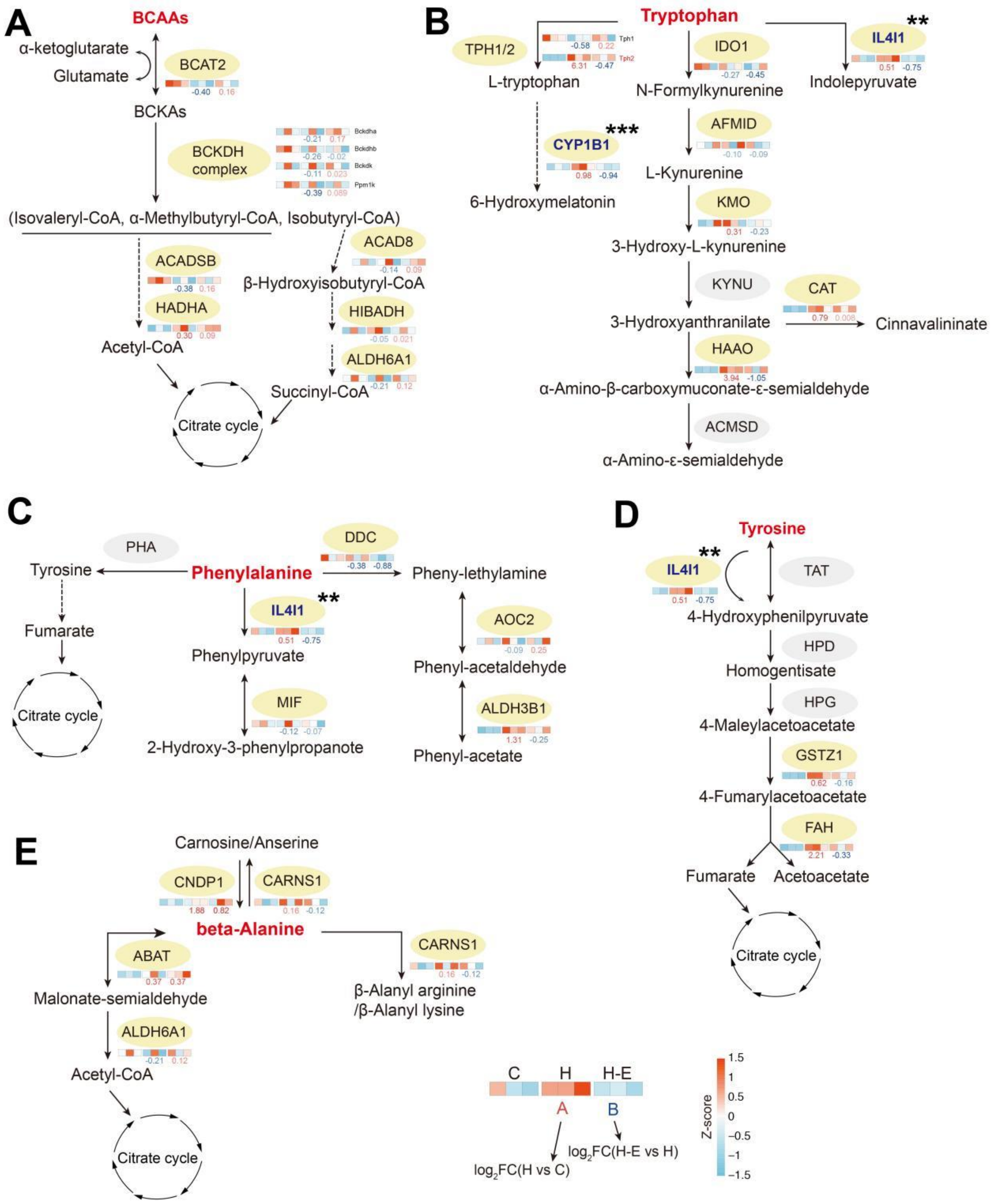

Supplementary Figure S4. Expression heatmap correlated to the metabolite-related pathways. The Z-scores between chow, HFD, and HFD-exercise groups on the mean gene expression level obtained from RNA-seq are shown in a blue–white–red scale. H vs C, HFD group versus chow group; H-E vs H, HFD-exercise group versus HFD group. $^{**}P < 0.01$ and $^{***}P < 0.001$.
